# Chronic Respiratory Diseases: The Stories You Don’t Want to Miss

**DOI:** 10.3390/medicina62040746

**Published:** 2026-04-13

**Authors:** Marcin Kurowski, Laura Malinauskienė, Corrado Pelaia

**Affiliations:** 1 Department of Immunology and Allergy, Medical University of Lodz, 90-419 Lodz, Poland; 2 Department of Pulmonology and Allergology, Vilnius University Hospital Santaros Klinikos, Santariskiu 2, LT-08661 Vilnius, Lithuania; 3 Department of Health Sciences, University “Magna Græcia” of Catanzaro, 88100 Catanzaro, Italy

Chronic respiratory disease is rarely boring. One day it is cilia that do not move; the next, it is an herbal tea that turns out to be an allergen, a life-saving drug that bites back, or lungs that will not quite forget a virus. This Special Issue of *Medicina* titled “Chronic Respiratory Diseases: Updates on Pathophysiology, Symptoms, Diagnosis and Treatment” brings these very different clinical stories under one roof and shows how closely they are connected by infection, immunity, and treatment.

The case report by Packi et al. [1] reminds us that even in patients with a well-defined chronic respiratory disorder, diagnostic thinking must remain wide open. A young woman with Kartagener syndrome and primary ciliary dyskinesia (PCD) developed persistent pleural effusion and eosinophilic pneumonia, initially treated as a bacterial process. Only after careful re-evaluation, recognition of marked eosinophilia, and targeted serology was pulmonary toxocariasis due to *Toxocara canis* confirmed, and the patient subsequently responded to albendazole.

This “first in the literature” co-occurrence of PCD and pulmonary toxocariasis highlights two key messages. First, structural or functional defects of mucociliary clearance profoundly reshape the lung’s infectious landscape, allowing usually trivial or transient pathogens—including parasites—to persist and trigger severe inflammation. Second, in any patient with chronic respiratory disease who does not improve on standard therapy, clinicians must resist anchoring bias and actively search for atypical causes, including parasitic infections, drug reactions, and autoimmune phenomena.

Preda and co-authors take us to the opposite end of the spectrum—from rare parasites to very common weeds [2]. Their review of IgE-mediated allergy to Asteraceae weed pollen and herbal medicinal products shows just how far-reaching ragweed, mugwort, and their relatives can be in everyday practice. They walk the reader through sensitization patterns; cross-reactivity with chamomile, marigold, echinacea, and milk thistle; and the sometimes dramatic reactions linked to “natural” remedies and bee products. The message is practical: structured diagnostics (skin tests, molecular allergology, challenge where needed) and good history-taking around herbal use are not optional extras, but part of modern respiratory and allergy care.

The theme of iatrogenic risk is taken up from a different angle by Mereškevičienė and Danila [3], who systematically review adverse effects of both first- and second-line antituberculosis drugs. As tuberculosis remains one of the leading infectious killers worldwide, multidrug and extensively drug-resistant strains necessitate prolonged multidrug regimens that are often toxic. The review synthesizes data on gastrointestinal, hepatic, neurological, cardiac, renal, and cutaneous reactions, identifies risk factors such as age, alcohol use, comorbidities, and acetylator status, and summarizes management strategies—from laboratory monitoring to dose adjustment, pyridoxine supplementation, and, inevitably, in some cases, treatment discontinuation.

Two points stand out. First, even the “old” drugs—isoniazid, rifampicin, pyrazinamide, ethambutol—still have safety profiles that are incompletely characterized in diverse real-world populations, underscoring the need for continued pharmacovigilance. Second, while new agents such as bedaquiline, delamanid, and pretomanid expand therapeutic options, they also introduce new patterns of toxicity and drug–drug interactions. Balancing a microbiological cure against the risk of serious adverse events is at the core of modern TB care, and this review offers a valuable, pragmatic guide for clinicians navigating that balance.

In another contribution to this Special Issue, Strumilienė et al. [4] provide a longitudinal window into what happens after the acute fight is over in severe and critical COVID-19. In a prospective cohort of 85 patients who were followed for 12 months after hospital discharge, they document gradual but incomplete recovery of spirometric parameters and diffusion capacity, with a substantial proportion of patients retaining restrictive patterns and impaired DLCO at one year. High-resolution CT scans reveal that while inflammatory changes diminish, profibrotic and fibrotic abnormalities remain strikingly prevalent.

Perhaps most intriguingly, the authors couple these structural and functional data with detailed immunophenotyping. They show sustained alterations in lymphocyte subsets, including reductions in CD3+, CD8+, and activated CD3HLA-DR+ cells, and identify associations between specific T-cell populations expressing CCR2 (CD192) and lung function indices—higher CD4+/CD28+/CD192+ counts correlate with worse total lung capacity, whereas higher CD8+/CD28+/CD192+ levels correlate with better outcomes. These findings reinforce the concept that long COVID is not merely “slow healing” but reflects ongoing immune dysregulation that can shape the trajectory of pulmonary repair and fibrosis.

What do these four contributions tell us when we read them side by side? First, they indicate that chronic respiratory diseases today sit at a crossroads where genetics, environment, infection, and treatment constantly intersect. Second, they make us think beyond classic bacteria and viruses and encourage us to question the safety of “natural” products in sensitized patients, respect the toxic potential of essential drugs, and follow our patients well beyond the acute phase of illness.

For clinicians, there is something immediately usable in each paper: a reminder to re-evaluate stubborn pneumonia, a framework for approaching “herbal” allergy, a structured approach to monitoring for TB drug toxicity, and concrete reasons to set up long-term follow-up for survivors of severe COVID-19. For researchers, these articles highlight priorities ranging from rare host–pathogen interactions to pharmacovigilance and immune mechanisms of repair.

A review by Janić et al. [5] takes the reader into the area of respiratory disease comorbidities. The glucagon-like peptide-1 (GLP-1) and glucose-dependent insulinotropic polypeptide/GLP-1 (GIP/GLP-1) receptor agonists have already gained wide recognition in several indications, including glucose control in type 2 diabetes and weight loss in people with and without diabetes. Janić et al. provide a comprehensive account of their potential influence on metabolic changes in the context of chronic obstructive pulmonary disease (COPD), asthma, pneumonia, obstructive sleep apnea, pulmonary hypertension, lung cancer, and lung transplantation. Given the known possible contribution of systemic inflammation associated with metabolic dysregulation to the development and progression of airway inflammatory conditions, this review is a must-read for all clinicians who are interested in updating their knowledge on systemic inflammation in respiratory disease.

Two articles in this Special Issue of *Medicina* address the role of sensitization to airborne allergens in the development of respiratory disease. A review by Klain et al. [6] highlights the role of *Alternaria* allergens in the development of respiratory allergy symptoms. In addition, the paper covers multiple aspects of allergen-specific immunotherapy for *Alternaria* allergy and provides insights into possible cross-reactivity between Alternaria allergens and those from other sources. Since mold allergy is an emergent and important, yet sometimes underestimated mechanism underlying respiratory allergy symptoms, this review is an important contribution to increasing awareness of mold spore allergy among allergy and respiratory practitioners. An original article by Majkowska-Wojciechowska and colleagues [7] explores patterns of reactivity to inhalant allergens in a central Polish population sample comprising over 1700 subjects. In the studied population, IgE sensitization to perennial allergens was significantly associated with asthma, whereas sensitization to seasonal airborne allergens, such as grass, rye, hazel, and alder pollen, was associated with the development of the symptoms of allergic rhinitis.

Last but not least, this Special Issue also contains articles addressing clinically important issues relating to cystic fibrosis (CF) [8], bronchiectasis [9], and respiratory failure in patients with chronic obstructive pulmonary disease (COPD) [8]. That last paper, by Ozdemir et al. [8], importantly identifies a higher comorbidity burden in females with COPD, along with heart failure and heart failure risk factors being more prominent in female COPD patients. A systematic review on the use of azithromycin in the management of CF in pediatric patients with or without concomitant *P. aeruginosa,* provided by Hassan Al-Shehri and Dana Albassam from Riyadh, Saudi Arabia [9], revealed widely beneficial effects of azithromycin in pediatric CF patients, leading to lung function improvement and considerable reduction in the number of exacerbations requiring antibiotic treatment. These effects of azithromycin contribute significantly to the improved efficacy of pediatric CF management and justify its use in pediatric CF. Finally, the research article by Janković and colleagues [10], from Belgrade, Serbia, explores differences in clinical characteristics, presentation, severity, or distribution in focal and multifocal bronchiectasis, as well as their prognostic implications. The bottom line of their article is that the presence of multifocal bronchiectasis may serve as a biomarker of disease severity and poor outcomes.

The wide range of topics addressed by the contributors to this Special Issue evidently reflects the heterogeneity of clinical challenges that most pulmonologists and allergists face almost every day when managing patients with respiratory conditions. Addressing these challenges may be facilitated by guidelines and recommendations issued by international scientific groups with regard to asthma [11], COPD [12], tuberculosis [13], COVID-19 [14], cystic fibrosis [15], bronchiectasis [16], and primary ciliary dyskinesia [17] within comparable frames.

IgE-dependent allergen sensitization is an important factor in the development of type 2 inflammation in allergic respiratory diseases, such as asthma and allergic rhinitis. Recent advances in molecular biology and component-resolved diagnostics (CRD) of allergy have improved the accuracy of identifying the culprit allergenic proteins and enabled more detailed recommendations based on the sensitization profile [18]. This is particularly applicable to the identification of possible cross-reactivity between airborne and food allergens, which may be vital for preventing anaphylactic reactions [19,20,21,22,23,24,25,26,27]. Molecular identification of sensitizing allergen components is also a valuable tool for a detailed assessment of inhalant allergy [28,29,30,31,32] and facilitates decisions regarding allergen-specific immunotherapy, increasing the possibility of successful outcomes [33,34,35,36,37].

Last but not least, a wide and optimistic perspective in respiratory disease management opens up with the implementation of biological treatment. Biologics are increasingly becoming the mainstay of treatment for severe asthma [38,39,40,41] and chronic rhinosinusitis with nasal polyps [42,43,44], while recent developments in biologics for COPD [45,46,47] raise hopes for substantial benefits for patients with other respiratory conditions as well.

If you look after lungs—in the clinic, in intensive care, in a TB ward, or in an allergy lab—this Special Issue is an invitation to pause, take a breath, and read further. Each article presents a different story, but together, they sketch a single, evolving picture of what it means to live with and treat chronic respiratory disease today. We hope to have convinced you to read the articles in the Special Issue that we have had the pleasure and honor of co-editing.
